# White Matter Alterations in Depressive Disorder

**DOI:** 10.3389/fimmu.2022.826812

**Published:** 2022-05-12

**Authors:** Enling He, Min Liu, Sizhu Gong, Xiyao Fu, Yue Han, Fang Deng

**Affiliations:** Department of Neurology, The First Bethune Hospital of Jilin University, Changchun, China

**Keywords:** major depressive disorder, pathogenesis, white matter alterations, clinical relevance, depression, diffusion tenor imaging

## Abstract

Depressive disorder is the most prevalent affective disorder today. Depressive disorder has been linked to changes in the white matter. White matter changes in depressive disorder could be a result of impaired cerebral blood flow (CBF) and CBF self-regulation, impaired blood-brain barrier function, inflammatory factors, genes and environmental factors. Additionally, white matter changes in patients with depression are associated with clinical variables such as differential diagnosis, severity, treatment effect, and efficacy assessment. This review discusses the characteristics, possible mechanisms, clinical relevance, and potential treatment of white matter alterations caused by depressive disorders.

## Introduction

A depressive disorder, an affective disorder, is defined by significant and persistent depression. It is frequently accompanied by a variety of cognitive impairments and somatic symptoms. Depressive disorders are classified as disruptive mood disorder, major depressive disorder (MDD), persistent mood disorder (PSD), transitory depressive disorder (TSD), persistent mood disorder, premenstrual dysphoric disorder, substance/medication-induced depressive disorder, other disease-induced depressive disorder, other specified depressive disorder, and unspecified depressive disorder in the Diagnostic and Statistical Manual of Mental Disorders-5 (DMS-5). International studies revealed a significantly higher prevalence of depressive disorders in people with white matter lesions (WMLs) than in health (1–3). Additionally, the correlation between white matter alterations in various parts of the brain and depressive disorder is different (4). Therefore, this review will focus on the white matter changes associated with MDD and their research progress.

## Possible Pathogenesis of White Matter Changes Caused by MDD

The white matter is made up of nerve fibers that have aggregated. Most of the nerve fibers are myelinated. Thus, white matter injury primarily affects the myelin sheath of nerve fibers. It is easily influenced by a variety of factors, including ischemia, hypoxia, and inflammatory stimulation. The pathogenesis of white matter changes in MDD is still unclear. According to current research, it may be related to cerebral blood flow regulation and cerebral blood flow self-regulation, blood-brain barrier damage, inflammatory factors, genetic and environmental factors.

### Cerebral Blood Flow and Cerebral Blood Flow Self-Regulation

Appropriate cerebral blood flow (CBF) is required to maintain the brain’s structural and functional integrity. In health, self-regulation of CBF can help maintain a constant CBF (5). Reduced CBF, on the other hand, does not have to result in ischemia to influence brain function. Mild CBF insufficiency may impair cognitive and emotional function (6). White matter in the terminal area of the deep perforating artery blood supply has a lower capillary density than the cortex between adjacent small arteries (7). Hence, white matter is more susceptible to insufficient CBF. In comparison to the control group, those with a depressive disorder had less white matter CBF in the bilateral anterior cerebral artery regions and the left middle cerebral artery region (8). Increased white matter hyperintensity (WMH) was associated with decreased CBF in the rostral anterior cingulate cortex volumes in late-life depression, according to an MRI study (9). Colloby et al. discovered that when participants’ depression was in remission, white matter CBF was significantly higher than in the control group, but not in patients with current depression (10). It demonstrates the point in the opposite direction.

CBF self-regulation occurs *via* three primary mechanisms: cerebral autoregulation (CA), cerebrovascular reactivity (CVR), and neurovascular coupling (NVC). CA is a mechanism that rapidly stabilizes CBF by adjusting cerebrovascular resistance in response to changes in cerebral perfusion pressure. There are two types of cerebral autoregulation: static cerebral autoregulation (sCA) and dynamic cerebral autoregulation (dCA). CA was initially believed to be a static, stable process that contributed to slow, gradual blood pressure changes. Static cerebral autoregulation is primarily quantified using Single-Photon Emission Computed Tomography (SPECT) or Computed Tomography Perfusion imaging (CTPI). With the development of monitoring measurements, the term “dynamic autoregulation” was coined (11). Numerous methods for inducing blood pressure changes have been proposed to investigate dCA, including pharmacological means, thigh-cuff release, body tilt, handgrip, lower-body negative pressure, valsalva or sit-to-stand maneuvers, and transient carotid artery compression (12). However, this method is not appropriate for all people. With many mathematical methods proposed, it is possible that induced changes in ABP are not completely necessary (13). Transcranial Doppler (TCD) measures cerebral blood flow velocity (CBFV), which is a proxy for cerebral blood flow, while vascular unloading techniques in the fingers monitor changes in the finger’s blood pressure. Combining the two measurements is a widely used technique for determining the dCA because it is non-invasive, simple to implement, and cooperative. Luo et al. (14) also applied the same method to investigate the dCA’s role in depression. The study demonstrated that depression significantly impairs cerebral autoregulation, and that the higher the depression score, the more compromised CBF self-regulation is. Purkayastha et al. (15) then demonstrated a link between compromised cerebrovascular hemodynamics (pulsatility index and dCA) and white matter structural integrity. arteriosclerosis is one factor that contributes to decreased cerebrovascular reactivity. According to a cross-sectional study by van Sloten et al. (16), the degree of arteriosclerosis is directly proportional to the severity of depression, and a portion of the arteriosclerosis is associated with white matter lesions. These findings suggest that there may be a link between self-regulation of CBF and white matter changes in depressive disorder.

### Inflammatory Factors

Inflammatory factors play a critical role in the onset and progression of MDD. In comparison to healthy controls, MDD patients have higher levels of IL-6, tumor necrosis factor (TNF)-, IL-10, soluble IL-2 receptor, IL-13, C-C chemokine ligand-2, IL-13, IL-18, IL-12, IL-1 receptor antagonist, and soluble TNF receptor 2 in their peripheral blood (17). Increased inflammation is also associated with white matter damage. C-reactive protein, a marker of inflammation, is elevated in middle-aged people. The level is associated with a decrease in white matter changes related to MDD (18). Sugimoto et al. demonstrated that the fractional anisotropy (FA) values of the bilateral inferior fronto-occipital fasciculus (IFOF) and corpus callosum genu were significantly lower in MDD patients than in healthy controls and were negatively correlated with IL-1β levels (19). IL-1β can cause inflammation, resulting in cell death and damage in the central nervous system. Not only can IL-1β reduce myelination (20), but injection of IL-1β into the brain of newborn rats can decrease the number of developing oligodendrocytes, thereby causing direct myelination damage (21).

As a result, the increased level of inflammatory factors in the central nervous system of patients with MDD can result in inflammation, impairing the formation and integrity of the myelin sheath, ultimately resulting in white matter damage.

### Blood-Brain Barrier Damage

The blood-brain barrier (BBB) regulates material exchange and maintains the nervous system’s homeostasis due to its unique structure. An investigation carried out by Lesniak et al. (22) reveals that trauma destroys the BBB in mice. The material exchange function was out of control, resulting in nerve growth factor leakage. It exacerbated the mice’s depression-like behavior. Above all, MDD may be associated with the breakdown of the BBB structure. Chronic social stress, according to a study (23) using a mouse model of depression, has been shown to decrease the expression of claudin-5 in mice. It increases the BBB’s permeability and facilitates the entry of blood inflammatory factors such as interleukin (IL)-6 into the central nervous system. These factors contribute to the development of depression-like behavior in mice. Similarly, patients with MDD have decreased claudin-5 expression (24). MDD patients were found to have increased permeability because of an abnormal BBB structure. The research of Pearlman et al. (25) corroborated the preceding assertion. Normally, astrocytes S100β was found in the brain parenchyma. The study did discover, however, that S100β can be detected in the serum of MDD patients. Its level is significantly correlated with acute depressive symptoms fluctuations. Moreover, the breakdown of the BBB is corelated with white matter alterations. Kerkho et al. (26) used dynamic enhanced MRI to investigate patients with cerebral small vessel disease. They discovered that every 2 mm closer to WMLs, the change in parenchymal diffusivity increased by 0.01%. Additionally, the change in parenchymal diffusion over two years is positively correlated with the baseline permeability of the BBB. Indeed, it corroborates the hypothesis that the BBB is the cause of WMLs. The results of Topakian et al. (27) demonstrated that the normal white matter area exhibited increased permeability of the BBB. This demonstrates that the BBB is damaged prior to the white matter changes. Although there is no direct evidence that BBB disruption is associated with white matter changes in MDD patients. However, as previously stated, a destroyed BBB results in an excessive leakage of toxins such as inflammatory factors into the central nervous system, which can destroy the integrity of the white matter structure.

### Genes and Environmental Factors

A growing number of studies have established an association between certain psychiatric disorders and genetic and environmental factors (28, 29). MDD is one of them. The meta-analysis results of Sullivan et al. suggested that MDD has a heritability of 31% to 42% (CI=95%) (30). Methylation of the brain-derived neurotrophic factor (BDNF) promoter region has been shown to be negatively correlated with the FA value of the right radiated crown in patients with MDD (31). White matter structure is associated with genes, which are also found in MDD. A study of untreated patients with MDD showed damage to the brain white matter fiber bundles in the emotional control circuit. And it was associated with the TPH2-rs4570625 GG polymorphism (32). Furthermore, the serotonin transporter is involved in neuronal serotonin reuptake, which is the primary target of selective serotonin reuptake inhibitors. It is critical in MDD. Lesch et al. (33) demonstrated that the S allele of the 5HTTLPR polymorphism slows the serotonin transporter’s transcription in neurons. According to one default Diffusion Tensor Imaging (DTI) study, depressed elderly S-allele carriers have a lower FA than L homozygotes in frontolimbic brain areas such as the dorsal and rostral anterior cingulate, posterior cingulate, dorsolateral and medial prefrontal regions, and thalamus, among others (34). Moreover, won et al. (35) discovered a significant correlation between the white matter integrity of the corpus callosum and increased DNA methylation of the serotonin transporter SLC6A4 gene in MDD patients. It suggests a possible regulatory mechanism for gene-environmental interactions affecting white matter integrity in MDD. Although the well-known behaviorist psychologist John Broadus Watson’s “environmental determinism” overemphasizes the environment’s influence, the influence of environment on human growth and development cannot be ignored. A study on primates established that a poor feeding environment influences the integrity of white matter fiber bundles in the frontal lobes of Bonnet macaques (36). Witnessing domestic violence as a child is a traumatic experience that increases one’s risk of developing depression (37), post-traumatic stress disorder (38), and other affective disorders. The FA value of the left suboccipital longitudinal tract was significantly lower in adolescents who witnessed domestic violence than in the control group, and was negatively correlated with the depression and anxiety scale scores (39). However, Meinert et al. argued that damage to the white matter tracts in MDD was caused by childhood abuse, not by the MDD diagnosis itself (40).

Not only can the pathogenesis exist independently, but they can also interact. For instance, insufficient CBF results in white matter ischemia. Then oxidative stress can occur, resulting in an increase in reactive oxygen species. Furthermore, reactive oxygen species can result in the breakdown of the blood-brain barrier, promote white blood cell migration, and enhance myelin phagocytosis. Additionally, it harmed the fragile central nervous system’s essential macromolecular substances by mediating cell damage (41). Finally, those factors caused continuous damage to the white matter. Simultaneously, ischemia and hypoxia of the white matter can activate astrocytes while activating the central protection mechanism. It induces an inflammatory response, aggravating the response to white matter injury

## White Matter Alterations in MDD

The advancement of imaging technology has made it possible to conduct non-invasive research on affective disorders, with Diffusion Weighted Imaging (DWI) playing a critical role in identifying brain abnormalities correlated with mental illness (42). In comparison, the majority of imaging examinations focus on macroscopic changes or functions, whereas the advancement and development of DTI, has shifted researchers’ attention to changes in the microstructure of the brain. It quantifies water molecule movement in the brain and is increasingly used to assess changes in the white matter microstructure. FA, which is the proportion of water molecules in the total dispersion tensor, can be used to determine the thickness of the myelin sheath and the degree of white matter damage. The Mean Diffusivity (MD) is a measure of the diffusion coefficient and serves as an indicator of the membrane’s density. The Axial Diffusivity (AD) is used to determine the number, diameter, and organization of axons; while the Radial Diffusivity (RD) provides additional information about the myelin sheath (43). Low FA and high MD values are both common manifestations of white matter changes associated with depression and other affective disorders (44). Many subsequent studies confirm the correlation between MDD and alterations in white matter.

### White Matter Fundamental Changes in MDD

White matter changes are also common in aging healthy individuals. Age-related white matter changes typically manifest as fuzzy linear, cap-shaped, or ring-shaped changes surrounding the ventricle or as multi-point and patch-shaped abnormal signals under the cortex (45). Rabins et al. (46) examined MDD patients aged 60 years or older. In comparison to the control group, MDD patients’ subcortical WMLs were more severe and contained more basal ganglia lesions. Nonetheless, the periventricular hyperintensity levels were comparable. As a result, periventricular hyperintensity may be unrelated to depression.

**Figure 1:**
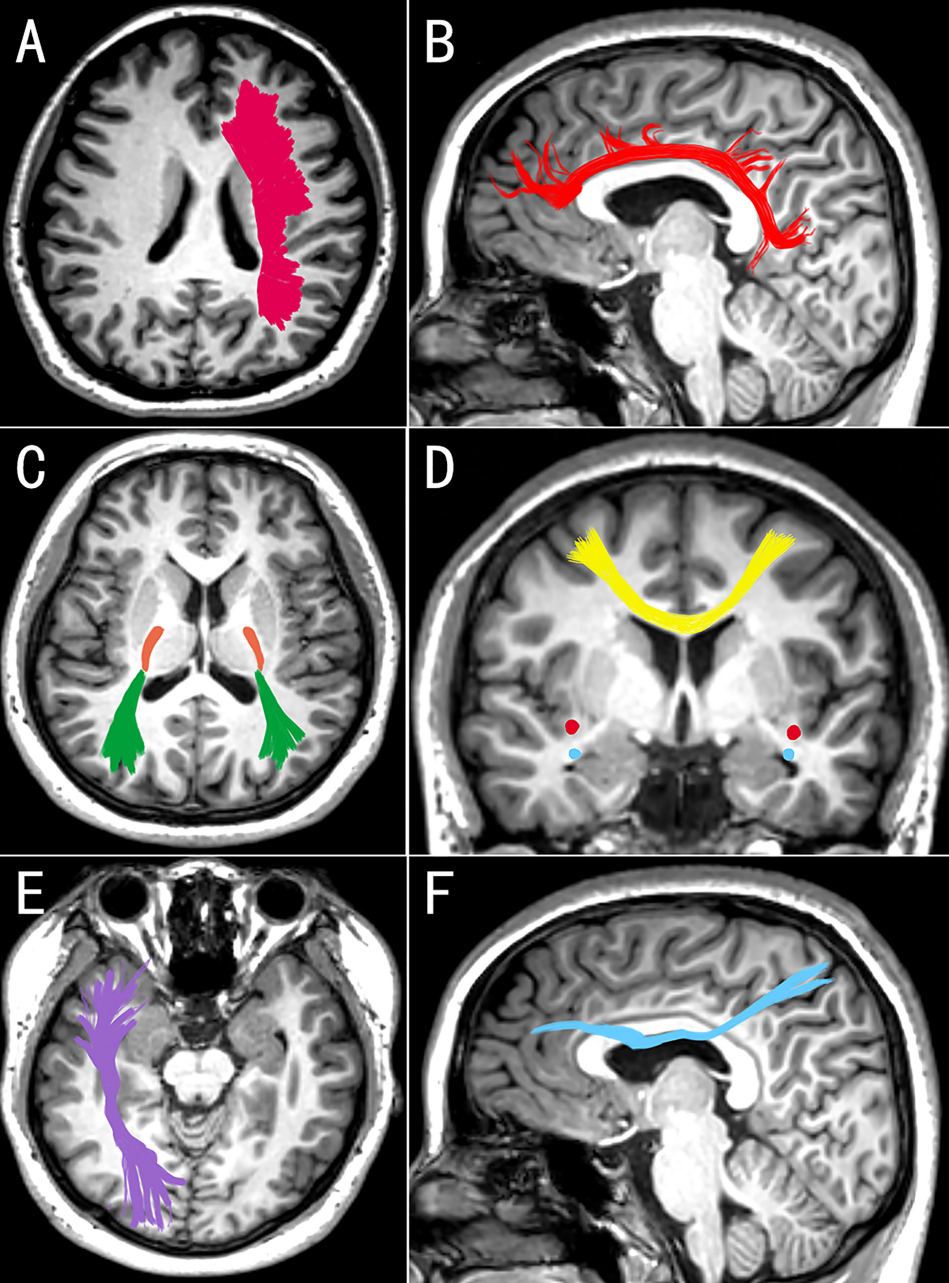
Images **(A, C, E)** are seen from above, medial image **(D)** is seen from behind, and images **(B, F)** are seen from the right. The white matter changes in corona radiata **(A)**, cingulum **(B)**, posterior thalamic radiation (**C**, green), posterior limb of the internal capsule (**C**, orange), corpus callosum (**D**, yellow), inferior fronto-occipital fasciculus (**C**, red), uncinate fasciculus (**D**, blue), inferior longitudinal fasciculus **(E)**, and superior longitudinal fasciculus **(F)** may be associated with MDD. These changes in white matter may be bilateral or unilateral (left or right) in different studies (Edited by Photoshop).

Neuroimaging techniques were used to map changes in white matter microstructure in MDD. According to the largest multicenter study to date, the DTI results for the MDD adult group revealed that the FA was lower in 16 of 25 interested white matter tracts than in the control group (47). It may reveal extensive structural connection defects in depression patients. The study of Cole et al. also supports this result (48). Among the damaged white matter, the corpus callosum and radiation crown were the most dissimilar parts of FA. Another meta-analysis of 641 depression patients and 581 normal controls revealed that depression patients had lower FA in the genu of the corpus callosum and the anterior limb of the internal capsule than the health (49). The callosum, as the largest inter-hemisphere association, connects the anterior cingulate cortex and orbitofrontal cortex of the two hemispheres and is critical for emotional regulation. Working memory and attention persistence are associated with the anterior cingulate cortex (50). The superior longitudinal fasciculus, inferior longitudinal fasciculus, fronto-occipital fasciculus, and posterior thalamic radiation have all been reported to have decreased FA of white matter (32, 51). As mentioned previously, the findings of several studies are inconsistent. The reason for the inconsistent findings could be the type and clinical phenotype of depression. Some scholars questioned the association between white matter FA and depressive symptoms. Dillon et al. found that anhedonia is negatively correlated with the FA in the genu, cingulum, and uncinate fasciculus and positively correlated with radial diffusivity (52). Affected white matter pathways connect the critical regions for value coding (53). In depression, Coloigner et al. discovered a positive correlation between anxiety and the FA of the genu and splenium of the corpus callosum, the anterior corona radiata, and the posterior thalamic radiation (54). As a result, the inconsistent findings may be explained by the interference of various clinical symptoms. The characteristics of white matter changes vary between different MDD groups. Late-onset depression primarily impairs the integrity of fiber tracts in the frontal lobe system (55). Compared with other types of depression, executive function is significantly impaired, white matter microstructure is significantly altered, and the incidence is higher (56, 57). Postpartum depression primarily affects the left anterior limb of the internal capsule. This change is related to the severity of MDD (58). Furthermore, Zhang et al. (59) discovered that functional connectivity within white matter bundles and between white matter and gray matter was decreased in MDD patients. The internal capsule’s functional connectivity with the cingulate cortex is the most severely damaged. To summarize, patients with MDD not only have structural damage to their white matter, but also have some functional impairment (**Table 1** and **Figure 1** provide the summary of white matter alterations in MDD in this review).

**Table 1:** The summary of white matter alterations in MDD in this review.

Study	Group	IFOF	CC			UF	Corona Radiata		PTR	SLF	ILF	ALIC	CCG	Main findings
			genu	body	splenium		ACR	PCR						
Sugimoto et al., 2018 (19)	35 drug-naive MDD patients; 35 HCS	√	√											White matter changes in IFOF and gCC are associated with MDD.
Choi et al., 2015 (31)	60 MDD patients; 35 HCS		√				√	√	√					Compared with the control group, MDD group found lower FA in gCC, the bilateral ACR, PCR, and the bilateral PTR.
Ping et al., 2019 (32)	118 first-episode, medication-naïve, MDD patients; 118 HCS		√	√			√							In the MDD patient group, the FA values in the genu and body of CC and the bilateral ACR were significantly reduced in the MDD patient group. White matter changes in MDD may be associated with the TPH2-rs4570625 GG polymorphism.
Won et al., 2016 (35)	35 medication-naive MDD patients;49 HCS		√	√										MDD patients had significantly lower FA values for the genu and body of the CC, compared with the control group. The FA and AD values in the body of the CC correlated with SLC6A4 DNA methylation.
van Velzen et al., 2020 (47)	1305 MDD patients; 1602 HCS		△				△							The FA was lower in 16 of 25 interested white matter tracts than in the control group. Among the damaged white matter, the CC and corona radiata were the most dissimilar parts of FA.
Cole et al., 2012 (48)	66 recurrent MDD patient; 66 HCS		△				√			√				Extensive structural connection defects in MDD patients, including regions in the CC, SLF and ACR, compared with the control group.
Chen et al., 2016 (49)	641 MDD patients; 581 HCS		√	√								√		The MDD group had significantly reduced FA values for the genu and body of the CC and ALIC, compared with the HCS.
Liao et al., 2013 (51)	231 MDD patients; 261HCS	√	√	√					√		√			The main fascicles involved were the right ILF, right IFOF, right PTR and interhemispheric fiber running through the genu and body of the CC.
Dillon et al., 2018 (52)	38 unmedicated adults with MDD; 52HCS		√			√						√	√	Decreased FA in the genu of CC , UF, PLIC and CCG reported in the depressed patients.
Coloigner et al.2019 (54)	114 MDD patients; 65HCS		√		√		√		√					Increased anxiety in MDD was related with greater FA values in gCC and sCC, ACR and PTR.

IFOF, inferior fronto-occipital fasciculus; CC, corpus callosum; gCC, genu corpus callosum; sCC, splenium of the corpus callosum; UF, uncinate fasciculus; ACR, anterior corona radiata; PCR, posterior corona radiata; PTR, posterior thalamic radiation; SLF, superior longitudinal fasciculus; ILF, inferior longitudinal fasciculus; PLIC, posterior limb of the internal capsule; CCG, cingulum. HCS, healthy control; FA, fractional anisotropy; AD, axial diffusivity. The √ indicates the affected tract. The △ indicates that involved parts of CC or corona radiata are not indicated.

### The Effect of MDD on Cognition and Its Relationship With White Matter Abnormalities

Previous Studies have shown that cognitive dysfunction is common in MDD patients and may persist in the period of remission (60, 61). Residual cognitive impairment may adversely affect occupational and social function (62). A variety of cognitions are impaired in MDD, such as set-shifting, processing speed, memory, learning, and verbal fluency (63, 64). And decreased executive function is the predominant cognitive manifestation. WMH is also correlated with cognitive impairment. Particularly, periventricular WMH associated with executive function and processing speed (65). Herrmann et al. illustrated that late-onset depression mainly impaired executive function and processing speed (56). There may be a potential link between white matter alterations and cognitive impairment in patients with depression (66). Compared with the control group, MDD patients showed significantly reduced FA in the right posterior cingulate cluster (PCC) which is positively correlated with performance in a verbal naming task (67). Moreover, Sheline et al. (68) reported that depression patients had greater WMH in the tracts of superior longitudinal fasciculus, fronto-occipital fasciculus, uncinate fasciculus, extreme capsule, and inferior longitudinal fasciculus. These white matter fibers connect the cortex associated with cognition and emotion functions. Hence, structural dysconnectivity between regions of gray matter may be the source of cognitive changes in depressed patients. Superior longitudinal fasciculus, fronto-occipital fasciculus and extreme capsule are the part of dorsolateral Prefrontal circuit that connects the dorsolateral prefrontal cortex and the basal ganglia. Impaired dorsolateral prefrontal circuits in MDD patients present the executive dysfunction, such as working memory, set switching, and inhibitory control (54, 69). Additionally, the response to happy faces is reduced in right fusiform activation (70), which may result from white matter disruption in this region (51). A study on late life depression demonstrated that the poorer attentional set-shifting, slower processing speed, and dysexecutive behavior is associated with the structural dysconnectivity of cognitive-control, sensorimotor, and attentional networks caused by WMH (71). Therefore, we consider that cognitive impairment in MDD may be the result of the structural dysconnectivity of multiple functional networks. The relationship between depression and cognitive dysfunction is still not fully understood, and whether it is cause, effect or interaction is still not fully unified (72). Hence, the above content only discussed the impact of depression on cognition.

### The Relationship Between White Matter Changes and MDD Clinical Relevance

The changes in the white matter of patients with MDD are highly correlated with clinical practice. It provides new concepts for diagnosing psychiatric disorders and relates to a variety of factors, including differential diagnosis, severity evaluation, quantification of treatment effects, and prognosis assessment.

Bipolar Depression (BD) and Schizophrenia (SZ) are both well-known mental illnesses. Depression is frequently the only symptom in the early stages of both diseases (73). Therefore, effective methods for identifying them are required. Significant differences in the limbic system were discovered between schizophrenia/bipolar disorder and major depressive disorder in large-scale meta-analyses (74, 75). Additionally, the callosal, limbic-paralimbic-hetermodal, cortico-cortical, thalamocortical, and cerebellar white matter integrity is significantly altered in the bipolar disorder and schizophrenia groups. In these areas, the MDD group, in contrast to the bipolar disorder and schizophrenia groups, exhibits no significant changes in white matter integrity. According to the preceding contents, MDD can be distinguished from bipolar disorder and schizophrenia based on the severity and extent of white matter changes.

MDD severity is associated to the location of white matter changes. Zhu et al. (76) demonstrated a negative correlation between the FA in the left anterior limb of the internal capsule and the severity of depression. However, in patients with late-onset depression, the severity of the condition is significantly associated with the microstructural changes in the left superior longitudinal fasciculus and the right uncinate fasciculus (77). Therefore, different types of depression may have distinct white matter regions that can be used to assess depression severity. Additional detailed analysis is required prior to clinical application.

Many studies have also been conducted on white matter changes in the treatment effect and prognosis evaluation of MDD. Emotional fluctuations have an effect on the integrity of white matter (78). A study comparing elderly depression patients before and after a year using DTI indicated that patients who did not achieve remission had a lower FA value for white matter in the anterior cingulate cortex than patients who did achieve remission or the control group (79). Then, some researchers examined changes in the white matter of MDD patients before and after treatment. Pillai et al. reported that the FA values between the raphe and left amygdala of patients who were not cured were significantly higher than those of cured patients (80). Accordingly, the integrity of the white matter connections within the emotional regulation network may be a critical characteristic of effective antidepressant treatment (81). Prefrontal and amygdala/hippocampal white matter connectivity has been demonstrated to be associated with antidepressant treatment response (82). Moreover, alterations in white matter are associated with the persistence and recurrence of depression (83).

## Potential Therapies

As previously stated, white matter changes in MDD are associated with depressive symptoms, and some are reversible. They provide us with a novel method of treating depression. It is known that antidepressant medications work by increasing the expression of neurotrophic factors such as BDNF, increasing the availability of serotonin and norepinephrine, and exerting anti-inflammatory effects. Antidepressant medications have been shown to improve white matter volume (84). Additionally, Wang et al. demonstrated that desvenlafaxine protects white matter from injury caused by stress (85). Cilostazol, phosphodiesterase-3, is an antiplatelet agent inhibitor. recently, phosphodiesterase is being used in the treatment of affective disorders. Phosphodiesterase regulate the intracellular levels of cyclic adenosine monophosphate (cAMP) and cyclic guanosine monophosphate (cGMP) *via* hydrolyzing cyclic nucleotides (86). Increased cAMP may stimulate transcription of the cAMP response element-binding protein (87, 88), thereby increasing BDNF expression (88). BDNF is involved in the regulation of neuronal pathways and synaptic plasticity. According to the research of Khadivi et al., the HAMD score decreased significantly more in MDD patients treated with cilostazol and sertraline for six weeks than in those treated with sertraline alone, and the safety was no difference (89). When Cilostazol combined with escitalopram, similar results were obtained (90). A case report found that cilostazol was used intensively to treat a patient with intractable depression complicated by WMH. The comparison of before and after treatment data revealed an improvement in the patient’s depressive symptoms and CBF to WMH regions (91). Due to the small sample size, however, the potential role of cilostazol in MDD patients with WMH requires further investigation.

Exercise also has a similar molecular mechanism with antidepressant drugs to improve depression symptoms (92). A study examined the effects of exercise training (stationary bicycle) on healthy and schizophrenic patients over a six-month period. They discovered that regardless of the psychiatric diagnosis, exercise significantly improves white matter integrity (93). Therefore, it is reasonable to consider whether exercise also influence white matter integrity in MDD.

Antidepressant medications indirectly repair white matter *via* a variety of molecular mechanisms. Repetitive transcranial electrical stimulation (rTMS) is a relatively new treatment modality that is primarily used in the treatment of major depressive disorder (MDD) and treatment-resistant depression. Even though the underlying mechanism for treating MDD remains unknown, one proposed mechanism involves directly altering abnormal brain functional and structural connectivity (94). An abnormal frontier-limbic network is the root of emotion regulation disorder (49). Studies have established that rTMS significantly alters the neural activity in fronto-limbic brain regions in MDD patients (95, 96). Additionally, a meta-analysis found that rTMS was more likely to induce remission (97).

## Conclusion

Many studies have established that white matter changes are characteristic of MDD. Moreover, those changes in MDD patients are related to a variety of factors, including differential diagnosis, the severity of MDD, treatment effect evaluation, and prognosis assessment. Hence, we can use and develop imaging methods to provide patients with a more objective diagnosis and comprehensive evaluation, as well as an individualized treatment plan to enhance the treatment effect and prognosis

## Author Contributions

EH conceived the review, collected the data, wrote the paper. ML helped analyze the data. SG, XF, and YH revise the manuscript. FD supervised the review and approved the final version of the manuscript. All authors contributed to the article and approved the submitted version.

## Funding

This work was supported by a grant from the National Natural Science Foundation of China (82071293).

## Conflict of Interest

The authors declare that the research was conducted in the absence of any commercial or financial relationships that could be construed as a potential conflict of interest.

## Publisher’s Note

All claims expressed in this article are solely those of the authors and do not necessarily represent those of their affiliated organizations, or those of the publisher, the editors and the reviewers. Any product that may be evaluated in this article, or claim that may be made by its manufacturer, is not guaranteed or endorsed by the publisher.
